# A proteoliposome formulation derived from *Bordetella pertussis* induces protection in two murine challenge models

**DOI:** 10.1186/1471-2172-14-S1-S8

**Published:** 2013-02-25

**Authors:** Sonsire Fernández, Esther M Fajardo, Aleida Mandiarote, Gemma Año, Maria A Padrón, Michel Acosta, Rubén A Cabrera, Luis A Riverón, Maydelis Álvarez, Kirenia Blaín, Mildrey Fariñas, Daniel Cardoso, Luis G García, Concepción Campa, José L Pérez

**Affiliations:** 1 Research & Development Vice-presidency, Finlay Institute. Havana, Cuba

## Abstract

Whooping cough remains a health problem despite high vaccination coverage. It has been recommended that development of new strategies provide long-lasting immunity. The aim of this work was to evaluate the potential of proteoliposomes (PL) extracted from *Bordetella pertussis* as a vaccine candidate against whooping cough. The size of the *B. pertussis* PL was estimated to be 96.7±50.9 nm by Scanning Correlation Spectroscopy and the polydispersity index was 0.268. Western blots using monoclonal antibodies revealed the presence of pertussis toxin, pertactin, and fimbriae 3. The Limulus Amebocyte Lisate (LAL) assay showed endotoxin levels lower than those reported for whole cell pertussis licensed vaccines, while the Pyrogen Test indicated 75 ng/mL/Kg. The PL showed high protection capacity in mouse challenge models. There was 89.7% survival in the intracerebral challenge and total reduction of the number of CFU in the intranasal challenge. No significant differences (p>0.05) were observed between mice immunized with *B. pertussis* PL and the Cuban DTwP vaccine, whichever challenge model used. These results encouraged us to continue the development of the *B. pertussis* PL as a component of a new combined vaccine formulated with tetanus and diphtheria toxoids or as a booster dose for adolescents and adults.

## Introduction

Whooping cough remains an important cause of public health concern worldwide. Data from WHO revealed that more than 16 million cases and about 195 000 deaths were reported in 2008 [1]. Despite high vaccination coverage with whole-cell (wP) or acellular (aP) vaccines, pertussis is still prevalent, probably in part, as a result of waning immunity and for that reason new vaccine strategies, able to provide long-lasting immunity have been recommended [2]. Bacterial-derived proteoliposomes (PL) are nanoparticulate vesicular structures that contain proteins, lipids and native lipopolysaccharide (LPS). To date, only a few licensed PL-based vaccines are available. One of them is VA-MENGOC-BC®, a vaccine composed of PL obtained from the outer membrane of *Neisseria meningitidis* serogroup B. This PL has been shown to have high efficacy in controlling meningococcal disease [3] and has also exhibited potent adjuvant activity when used with other vaccine antigens not only in its native form, but in cochleate form too [4]. Thus, a PL derived from *B. pertussis* could be a good approach for a new candidate vaccine against whooping cough.

## Materials and methods

Cellular pellets of *B. pertussis* strain 165 grown at industrial scale, inactivated with formalin 0.1% through 48-56 hours, were homogenized with 30 mM Tris buffer, containing 2mM EDTA, pH 8.5 at a ratio of 100-200mg/mL. Sodium deoxycholate (Fluka, Switzerland) was added at a ratio of 0.1-1 mL/g of biomass. The mixture was incubated for 1 hour and centrifuged at 33000g for 15 min. All supernatants were collected, subjected to a sequence of diafiltration processes and filtered using a Sartorius Minisart-plus unit of 0.2 µm. Particle size PL was determined by Photon Correlation Spectroscopy (PCS) (Beckman Coulter, Hialeach, FL, US). SDS-PAGE (polyacrylamide 13%) followed by a R250 Blue Coomassie or silver stain for LPS [5,6] was carried out using a low molecular weight marker (Amersham, UK). Western blot assays [7] were carried out using monoclonal antibodies (Mabs) against pertussis toxin (PT), pertactin (PRN) and fimbriae 3 (FIM3) (NIBSC, UK). The biological activity of the endotoxin present in the PL was determined by Limulus Amebocyte Lisate (LAL) [8] and by a Total Pyrogen Test in rabbits [9] at 10, 25, 50, 75 ng/mL concentrations of protein. Intracerebral challenge assays were carried out in 4-6 weeks old, 16-18 g, female OF1 mice (CENPALAB, Cuba) injected intraperitoneally with one dose of 0.5 mL of 60 µg of PL and aluminum hydroxide (1mg/mL), DTwP vaccine (Finlay Institute) prepared at 8 Ul/mL or placebo. Intracerebral challenge with *B. pertussis* 18323 (100-1000 DL_50_) was performed 2 weeks post immunization. All mice were observed for 14 days after the challenge and daily mortality was registered. For the intranasal challenge assay, BALB/c mice (female, 3-4 weeks, 12-14 g, CENPALAB, Cuba) were immunized subcutaneously with 2 doses, 125 µL separated by a three-week interval. Each dose consisted of 20 µg of PL and 0.25 mg of aluminum hydroxide. A group of mice was immunized with 125 µL of DTwP vaccine (Finlay Institute) while another group received 125 µL of PBS and aluminum hydroxide as placebo. The intranasal challenge and lung extraction was performed following the procedure described by Guiso *et al. *[10]. Results were expressed as the average Log _10_ of the CFU/g of lung for each group of mice at each extraction time after challenge.

### Statistical analysis

The Wilson approximate method and software R 2.10.0 [11] were used to calculate the confidence intervals. The arithmetic means ± standard deviation for Log _10_ CFU/ lung were also calculated. The comparison of arithmetic means of the groups was carried out by an analysis of simple variance (Statistic kit, Statgraphics Plus 5.0).

## Results and discussion

**Figure 1:**
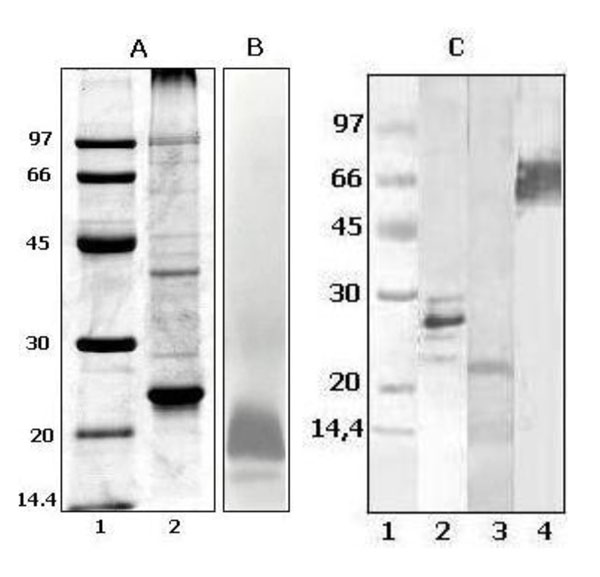
*B. pertussis* PL composition analysis; **Panel A,** SDS-PAGE (polyacrylamide 13 %) followed by R250 Blue Coomasie stain. Lane 1: Low molecular weight markers (97-14.4 kDa), Lane 2: *B. pertussis* PL. **Panel B,** SDS-PAGE of *B. pertussis* PL (polyacrylamide 13 %) followed by a LPS-specific silver stain. **Panel C,** Western blot of *B. pertussis* PL using Mabs or serum vs relevant antigens; Lane 1: Low molecular weight markers (97-14.4 kDa), Lane 2: PT anti serum (97/572), Lane 3: FIM 3 Mab (04/156), Lane 4: PRN anti serum (97/558).

Sodium deoxycholate was used to extract proteoliposomes from the outer membranes of inactivated cells of *B. pertussis* strain 165. The size of the PL was estimated to be 96.7 ± 50.9 nm with a polydispersity index of 0.268. SDS-PAGE (Figure 1 panels A and B) suggests the presence of molecules such as LPS and outer membrane proteins. Western blot assays using Mabs showed the presence of PT, FIM 3 and PRN (Figure 1 panel C), all of them are protective antigens present in acellular vaccines [12]. Proteomic and western blot studies with other Mabs are planed to completely elucidate the antigenic composition of *B. pertussis* PL. The biological activity of the endotoxin in PL was evaluated by the LAL method and showed a value of 4 960 EU/dose or 9 920 EU/mL. The endotoxic activity was higher than those reported for licensed aP vaccines (38 – 1 390 EU/mL), but lower than those reported for some licensed wP vaccines (11 400 – 181 100 EU/mL) [13], and similar to the meningococcal vaccine MenB (≤ 20 000 EU/mL) which consists of outer membrane vesicles [14]. In addition, the PL were non pyrogenic at concentrations less than or equal to 75 ng/mL/Kg of weigh of rabbits, according to the Total Pyrogen Test.

Mice immunized with both, the PL and DTwP vaccine, showed high protection levels against intracerebral challenge [89.7% and 91.2 of survival, respectively (95% confidence in the interval 76.4–95.9%)]. These values were significantly different in relation to the placebo group [12.1% of survival (95% confidence in the interval 4.8–27.3%)] and there were no differences between them (P<0.05). No mice died during the assay due to post-challenge trauma. Protective capacity induced by PL and DTwP vaccine, was also estimated by intranasal challenge (Table 1). Four days after challenge, no CFU were counted in lungs of mice immunized with PL or DTwP vaccine. Conversely, the number of CFU recovered from lungs of mice belonging to the Placebo group, at 4 and 7 days, was significantly (P<0.05) high compared with the other two groups. Overall, these results clearly indicate the potential of *B. pertussis* PL as a vaccine candidate against whooping cough.

**Table 1:** Intranasal challenge experiments in BALB/c mice using *B. pertussis* WHO reference strain 18323

Treatments (s.c. immunization)	Bacteria recovered post challenge (Log CFU/g lung ± S.D.)		
	
	2 hrs	4^th^ day	7^th^ day
**DTwP vaccine**	7.85 ± 0.39	NR	NR

***B. pertussis* PL**	6.68 ± 0.65	NR	NR

**PBS**	8.02 ± 0.2	6.85 ± 0.44	6.48 ± 0.11

NR. Not recovered

## Competing interests

The authors declare that they have no competing financial interests.

## Authors' contributions

SF conceived of the study, and participated in its design, carried out the purification of proteoliposomes, participated in the animal studies, and drafted the manuscript; EMF participated in the animal studies and helped in the revision of the manuscript; AM participated in the animal studies and in the analytical procedures; GA participated in the design of the study; MAP participated in the analytical procedures; MA participated in the analytical procedures; RAC participated in the analytical procedures; LAR participated in the purification of proteoliposomes; MA participated in the purification of proteoliposomes and in the animal studies; KB participated in the analytical procedures; MF participated in the animal studies; DC supplied the cellular biomass; LGG participated in the revision of the manuscript; CC participated in the revision of the manuscript; JLP conceived of the study, and participated in its design, carried out the purification of proteoliposomes, participated in the animal studies, and revised the manuscript.

## Declarations

This article has been published as part of *BMC Immunology* Volume 14 Supplement 1, 2013: Proceedings from Delivery Systems and Current strategies to drug design. The full contents of the supplement are available online at http://www.biomedcentral.com/bmcimmunol/supplements/14/S1.
